# Age Differences in Affective Experiences in Solitude: The Moderating Role of Nostalgia

**DOI:** 10.1093/geroni/igaf122.3045

**Published:** 2025-12-31

**Authors:** Xinyuan Peng, Jennifer Lay, Yuen Wan Ho, Dwight Tse, Helene Fung, Da Jiang

**Affiliations:** The Education University of Hong Kong, Hong Kong, China (People’s Republic); University of Exeter, Exeter, England, United Kingdom; Lingnan University, Hong Kong, China (People’s Republic); University of Strathclyde, Glasgow, Scotland, United Kingdom; The Chinese University of Hong Kong, Hong Kong, China (People’s Republic); The Education University of Hong Kong, Hong Kong, China (People’s Republic)

## Abstract

Solitude, defined as the absence of social interaction, is a common experience that increases with age. Research indicates that older adults enjoy solitude better than younger adults, influenced by gender, culture, and social networks. However, the role of nostalgia remains underexplored. Nostalgia, recalling time with close others, is a bittersweet emotion. Older adults experience nostalgia three times more frequently than younger adults. Based on socioemotional selectivity theory, older adults benefit from emotionally-meaningful goals. In accordance with the broaden-and-build theory of positive emotions, nostalgia correlates positively with positive emotions and well-being. This study investigated whether and how dispositional and daily nostalgia moderate associations between solitude, age, and affective experiences. Using the experience sampling method, participants (N = 188; Mage = 51.47, SDage = 17.75, range = 19-93, 66.50% women) completed smartphone-based assessments of momentary solitude, daily nostalgia, and positive (PA) and negative affect (NA) five times daily for seven consecutive days. Dispositional nostalgia was measured in a pretest. Results showed that nostalgia moderated associations between solitude, age, and affective experiences. For younger adults, higher dispositional nostalgia intensified the negative link between solitude and PA, while higher daily nostalgia amplified the positive link between solitude and NA. Nostalgia did not moderate the solitude-affect association in older adults. These findings challenge the typical view of nostalgia as a positive contributor to well-being and highlight its negative association with better affective well-being during solitude among younger but not older adults.

